# Supplementary material for: Potential of Hibiscus sabdariffa L. and Hibiscus Acid to Reverse Skin Aging
Source: Molecules. 2022 Sep 17;27(18):6076. doi: 10.3390/molecules27186076 (PMC9504376; doi:10.3390/molecules27186076)
Supplement: Supplementary file 1 [file molecules-27-06076-s001.zip › molecules-1912253-supplementary.pdf]

Potential of *Hibiscus Sabdariffa* Linn. and Hibiscus Acid to Reverse Skin Aging

Duanyang Wang[1], Maki Nagata[1], Masako Matsumoto[1], Yhiya Amen[2], Dongmei Wang[1], Kuniyoshi Shimizu[1]

Affiliation

[1] Department of Agro-Environmental Sciences, Graduate School of Bioresource and Bioenvironmental Sciences, Kyushu University, 744 Motooka, Nishi-ku, Fukuoka 819-0395, Japan

[2] Department of Pharmacognosy, Faculty of Pharmacy, Mansoura University, Mansoura 35516, Egypt

Correspondence

Assoc. Prof. Kuniyoshi Shimizu

Department of Agro-Environmental Sciences

Kyushu University

744 Motooka, Nishi-ku

Fukuoka 819-0395

Japan

Phone: +81 90-9491-4444

Fax: +81 92-802-4675

shimizu@agr.kyushu-u.ac.jp

SUPPLEMENTARY INFORMATION

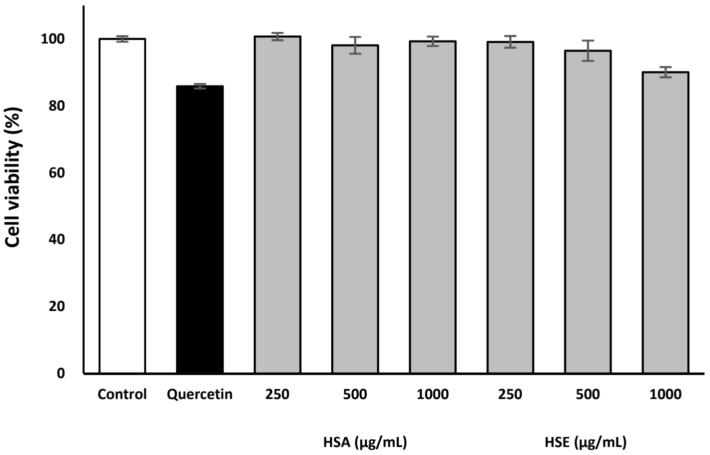

Supplementary Figure S1. RAW 264.7 cells were seeded in 96-well micro-plates at approximate density of 1 × 10^4 cells per well. After treatment with different concentration of HSA and HSE, the cell viability was measured by MTT assay. The control group was considered as 100%. HA groups against MilliQ control group because HA dissolved in MilliQ, and HE groups against DMSO control group because HE dissolved in DMSO. The results indicated that both HSA and HSE were no cytotoxicity to RAW 264.7 cells in the concentration below 1000 μg/ml.

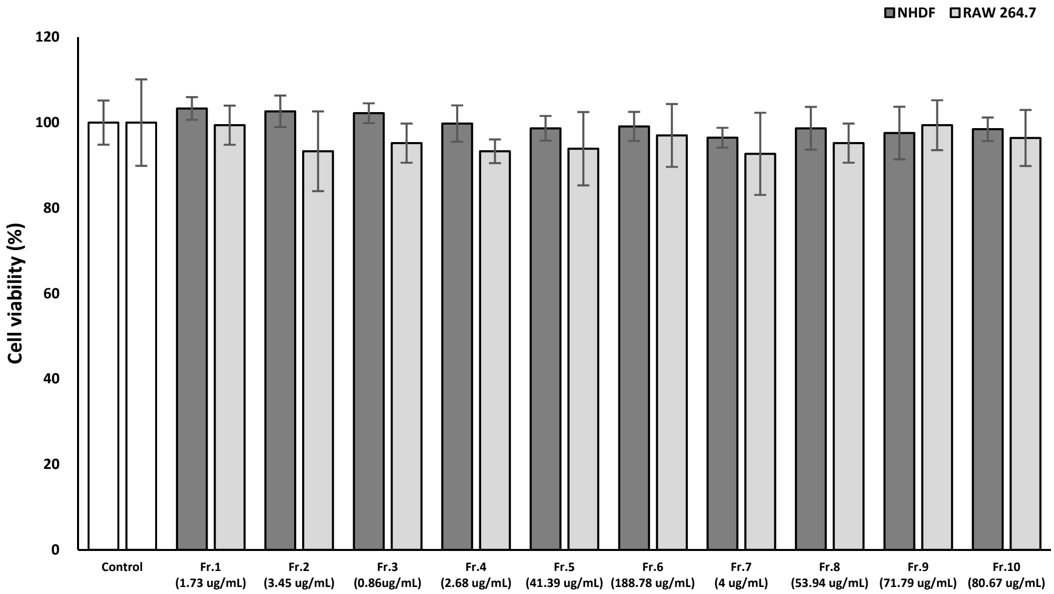

Supplementary Figure S2. The control group was considered as 100%. MTT results indicated that none of the 10 fractions had significant cytotoxicity to both NHDF cells and RAW 264.7 cells.

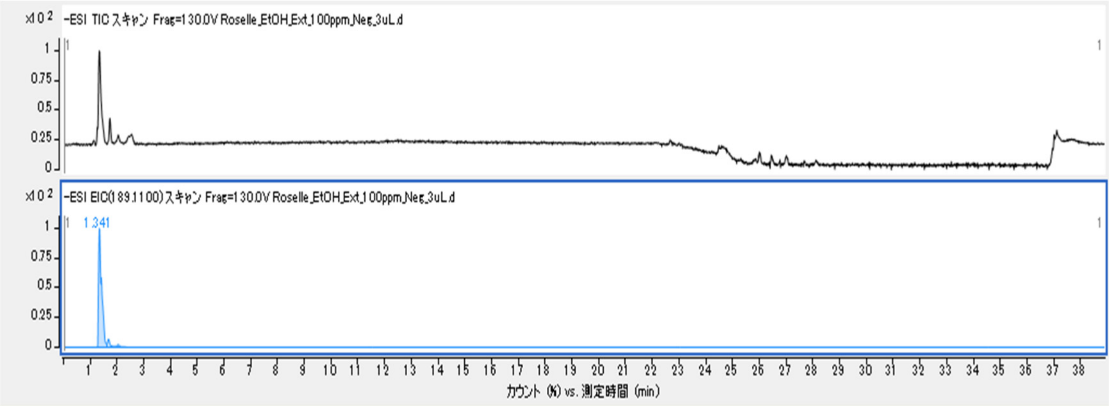

Supplementary Figure S3. LC/MS-qTOF analysis of HSE. Total ion chromatogram (TIC) spectrum of total HSE (upper) and extract ion chromatogram EIC of Hibiscus acid (lower).

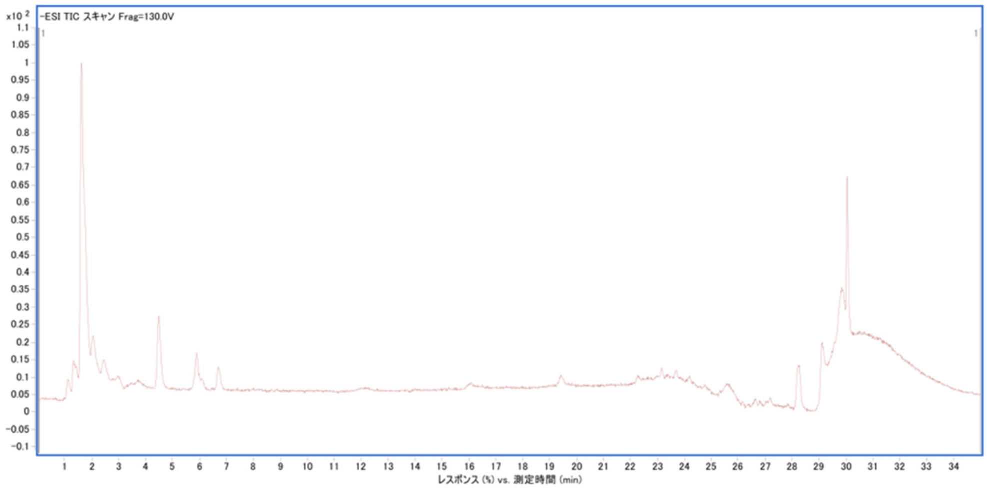

Supplementary Figure S4. TIC of Fr.5 by LC/MS-qTOF.

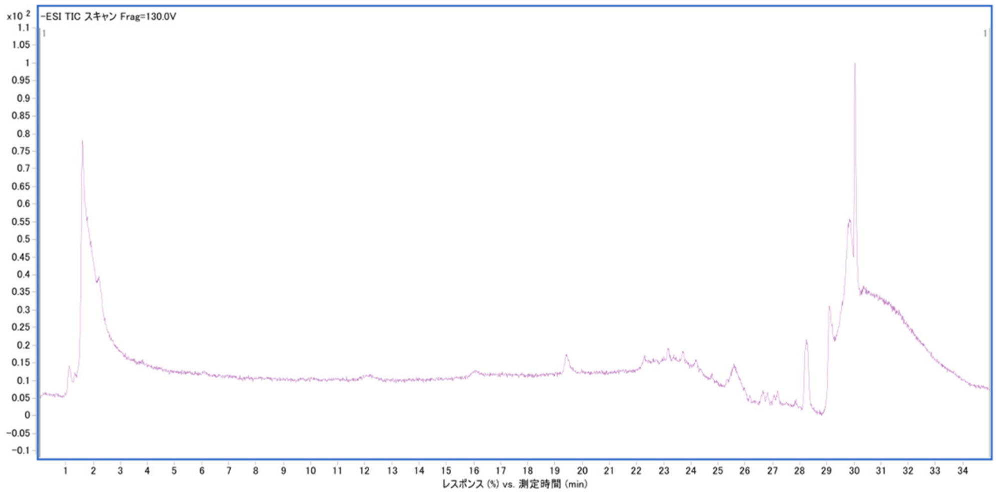

Supplementary Figure S5. TIC of Fr.6 by LC/MS-qTOF.

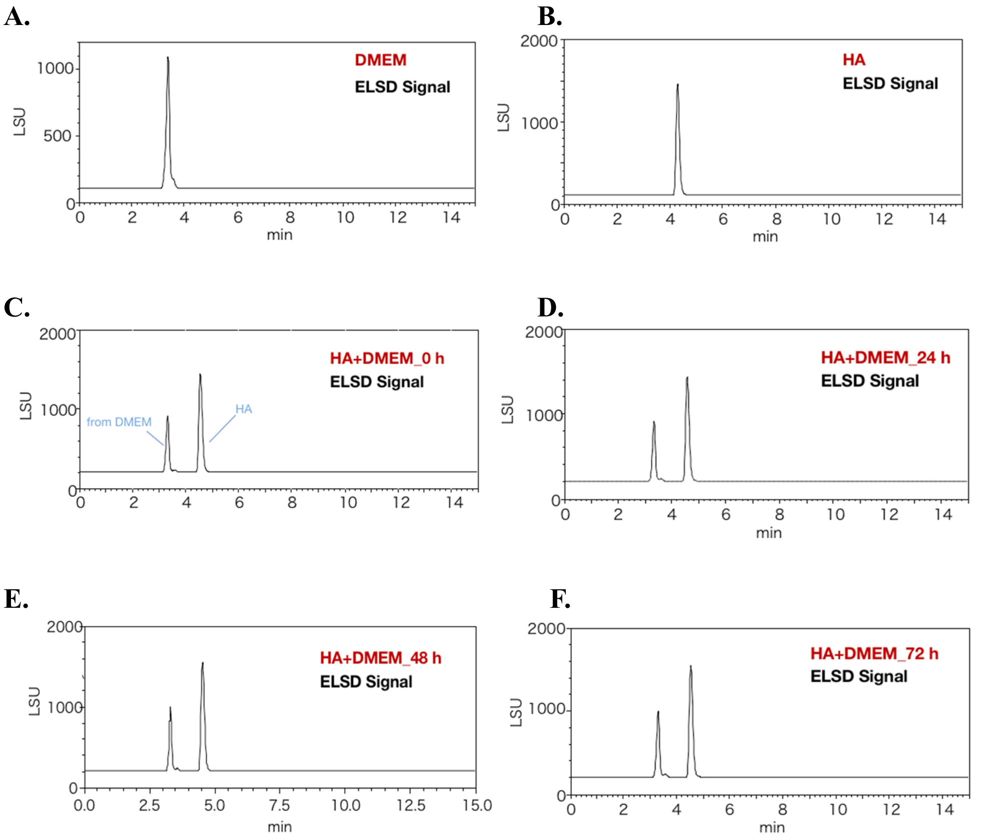

Supplementary Figure S6. Stability analysis of hibiscus acid (HA) in DMEM medium. HA was dissolved in DMEM, take 100 μL plus 3 times the volume of ACN and centrifugal for deproteinization, then filtration and injection. The final concentration was 5 mg/mL, and labeled as HA+DMEM 0 h group. Then 100 μL independently to three groups, incubated at 37 ℃ in a CO_2 incubator, after 24 h, 48 h, 72 h, same steps to deproteinization, filtration and injection. The final concentration was 5 mg/mL, and labeled as HA+DMEM 24 h, 48 h, 72 h groups. **A.** ELSD chromatogram of only DMEM. **B.** ELSD chromatogram of only HA. **C-F.** ELSD chromatogram of HA+DMEM 0 h, 24 h, 48 h and 72 h. The results indicated that the purity of the isolated HA compound reach to 99.7% and remained stable in DMEM.

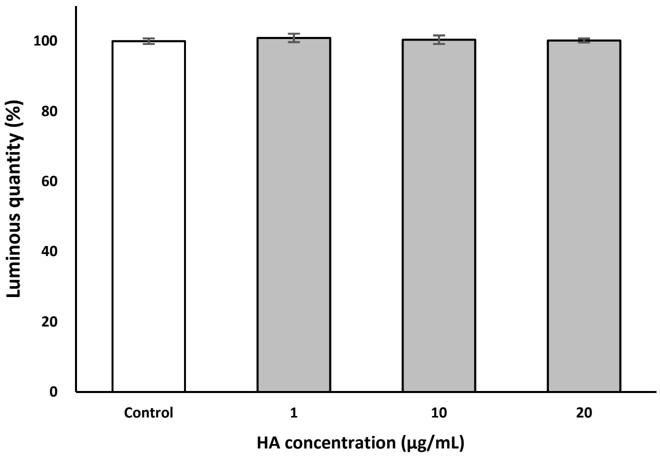

Supplementary Figure S7. The samples detected in the extracellular ATP assay were the supernatant of cell culture medium after HA treatment. Considering whether the sample would have an impact on the experiment results, same concentration HA were dissolved in the cell-free culture medium, and the luminescence was detected under the same conditions. The control group was 100% medium, and the result was considered as 100%. Results indicated that HA had no impact on experiment result.
